# Regional Brain Volumes Moderate, but Do Not Mediate, the Effects of Group-Based Exercise Training on Reductions in Loneliness in Older Adults

**DOI:** 10.3389/fnagi.2017.00110

**Published:** 2017-04-25

**Authors:** Diane K. Ehlers, Ana M. Daugherty, Agnieszka Z. Burzynska, Jason Fanning, Elizabeth A. Awick, Laura Chaddock-Heyman, Arthur F. Kramer, Edward McAuley

**Affiliations:** ^1^Department of Kinesiology and Community Health, University of Illinois at Urbana-ChampaignUrbana, IL, USA; ^2^Beckman Institute, University of Illinois at Urbana-ChampaignUrbana, IL, USA; ^3^Department of Human Development and Family Studies/Molecular, Cellular and Integrative Neurosciences, Colorado State UniversityFort Collins, CO, USA; ^4^Department of Health and Exercise Science, Wake Forest UniversityWinston-Salem, NC, USA; ^5^Office of the Provost, Northeastern UniversityBoston, MA, USA

**Keywords:** exercise, loneliness, aging, prefrontal cortex, amygdala, social support, stress, psychological

## Abstract

**Introduction:** Despite the prevalence of and negative health consequences associated with perceived loneliness in older adults, few studies have examined interactions among behavioral, psychosocial, and neural mechanisms. Research suggests that physical activity and improvements in perceived social support and stress are related to reductions in loneliness. Yet, the influence of brain structure on these changes is unknown. The present study examined whether change in regional brain volume mediated the effects of changes in social support and stress on change in perceived loneliness after an exercise intervention. We also examined the extent to which baseline brain volumes moderated the relationship between changes in social support, stress, and loneliness.

**Methods:** Participants were 247 older adults (65.4 ± 4.6 years-old) enrolled in a 6-month randomized controlled trial comprised of four exercise conditions: Dance (*n* = 69), Strength/Stretching/Stability (*n* = 70), Walk (*n* = 54), and Walk Plus (*n* = 54). All groups met for 1 h, three times weekly. Participants completed questionnaires assessing perceived social support, stress, and loneliness at baseline and post-intervention. Regional brain volumes (amygdala, prefrontal cortex [PFC], hippocampus) before and after intervention were measured with automatic segmentation of each participant's T1-weighted structural MRI. Data were analyzed in a latent modeling framework.

**Results:** Perceived social support increased (*p* = 0.003), while stress (*p* < 0.001), and loneliness (*p* = 0.001) decreased over the intervention. Increased social support directly (−0.63, *p* < 0.01) and indirectly, through decreased stress (−0.10, *p* = 0.02), predicted decreased loneliness. Changes in amygdala, PFC, and hippocampus volumes were unrelated to change in psychosocial variables (all *p* ≥ 0.44). However, individuals with larger baseline amygdalae experienced greater decreases in loneliness due to greater reductions in stress (0.35, *p* = 0.02). Further, individuals with larger baseline PFC volumes experienced greater reductions in stress due to greater increases in social support (−0.47, *p* = 0.02). No group differences in these pathways were observed.

**Conclusions:** The social support environment and resulting reductions in stress, as opposed to exercise mode, may represent important features of exercise programs for improving older adults' perceived loneliness. As amygdala volume has been linked to anxiety, depression and impaired cognitive control processes in the PFC, moderation findings suggest further investigation in this area is warranted.

**Trial Registration:** ClinicalTrials.gov identifier NCT01472744 (https://clinicaltrials.gov/ct2/show/NCT01472744?term=NCT01472744&rank=1).

## Introduction

The aging process is multifaceted, with the rate and magnitude of neural and cognitive declines varying between individuals. This suggests additional factors, such as physical and emotional health, shape aging trajectories. The experience of loneliness is one such factor that occurs in typical aging. Loneliness is generally higher during young adulthood, declines during middle age, and increases again in older adulthood. Unfortunately, the prevalence of loneliness among older adults over the age of 65 is estimated to be 7–20%, with 40% of older adults experiencing loneliness at least some of the time (Dickens et al., 2011; Ong et al., 2016). Loneliness is defined as a feeling of social isolation and is related to perceived inadequacies in the quality and quantity of one's social relationships (Russell et al., 1984; Coyle and Dugan, 2012). Because loneliness is multi-dimensional, even individuals with a large social network may feel socially isolated if the quality of social ties and support is perceived as unsatisfactory (Hawkley and Cacioppo, 2010). A considerable body of literature has connected loneliness to a host of health outcomes, including increased blood pressure and body mass index, lower inflammatory control, weakened immunity, poor sleep health, and mortality. Such declines in physical health have been linked directly to declines in cognitive ability during aging, and greater loneliness is associated with impaired cognition and increased rate of cognitive decline, reduced executive control, clinical dementia, and depression (Cacioppo et al., 2009; Coyle and Dugan, 2012; Shankar et al., 2013; Cacioppo and Cacioppo, 2014; Ong et al., 2016). Importantly, evidence suggests a dose-response relationship between loneliness and health in which chronically lonely individuals exhibit worse health outcomes when compared with individuals who report only situational or no loneliness (Hawkley and Cacioppo, 2007). Moreover, loneliness, and the factors that contribute to it, may be intervened upon to potentially slow cognitive decline and promote successful aging. As the aging population continues to grow rapidly, the burden loneliness poses to public health and our ability to intervene to improve older adults' quality of life may be significant.

The mechanisms by which loneliness influences health are complex and include psychological, emotional, behavioral, and neural processes (Ong et al., 2016). During older adulthood, increased feelings of loneliness often follow from decreases in social connectedness and increases in stress due to life transitions such as retirement, illness or disability, declines in physical mobility, or death of a spouse or loved one (Hawkley and Cacioppo, 2007; Aartsen and Jylhä, 2011; Masi et al., 2011). The stress buffering hypothesis posits that social support serves a protective health role during stressful events (Cohen, 2004; Hawkley and Cacioppo, 2007; Holt-Lunstad and Uchino, 2015). In other words, individuals with a strong and positive social support network are thought to be better able to cope with stress (Wethington et al., 2015). Stress appraisals and responses attenuate or exacerbate health both directly and indirectly through individuals' health behaviors. Older adults, when faced with stressful events, may be less likely to seek social support and employ active coping strategies when managing stress (Cacioppo et al., 2003), but may be more likely to use behavioral withdrawal as a coping mechanism (Hawkley and Cacioppo, 2007; Segrin and Passalacqua, 2010). As such, research suggests interventions to reduce loneliness should aim to enhance one's social environment and social interactions (Masi et al., 2011).

Physical activity programs represent one approach with promising evidence for reducing loneliness in older adults (McAuley et al., 2000; Masi et al., 2011). Although the benefits of physical activity are well-documented, activity levels decline with age (McAuley et al., 2009) and inactivity has been associated with social isolation (Reed et al., 2011). The combination of loneliness and decreased physical activity participation may have serious implications for older adults' physical and psychological health and quality of life. In a large sample of older adults aged 65 years and older, Netz et al. (2013) found that feelings of loneliness were highest among inactive participants, while individuals meeting federal guidelines for physical activity reported the lowest levels of loneliness. Similarly, McAuley et al. (2000) observed decreases in loneliness in older adults after a group-based, 6-month exercise intervention and reported that these improvements may have been partially explained by the social support environment of the program. Mackenzie et al. (2014) examined predictors of loneliness in 127 older adults enrolled in a 10-month aerobic exercise or stretching/toning program. While exercise condition and sex differentially predicted changes in loneliness, social support emerged as a stronger independent predictor. Further, individuals reporting higher physical activity levels, lower depression, and lower perceived stress also reported lesser loneliness across the intervention. The combination of the social support environment and physical exercise may, therefore, serve to reduce psychological distress, which may, in turn, reduce perceptions of loneliness (Masi et al., 2011; McEwen and Gianaros, 2011; McHugh and Lawlor, 2011).

Despite this evidence, the neural mechanisms through which physical activity, social support, and stress affect loneliness are largely unexplored (Ong et al., 2016). The equivocal body of literature on the “neurology of loneliness” (Cacioppo et al., 2014) suggests that perceptions of loneliness may be regulated by various brain regions, including the amygdala, prefrontal cortex (PFC), and hippocampus. These regions are central in determining behavioral and physiological responses to stress and, as such, may play particularly critical roles in older adults' experience of loneliness. Stress is known to induce amygdala growth while decreasing areas of the PFC and impairing neurogenesis in the hippocampus (McEwen, 2006; Davidson and McEwen, 2012). These changes in brain structure are associated with increased anxiety and depression (Davidson and McEwen, 2012) and reduced behavioral regulation (Hawkley and Cacioppo, 2010). For example, high stress events can trigger emotional regulation processes in the amygdala, inhibit cognitive control and attentional processes in the PFC, and impair memory functions in the hippocampus (McEwen, 2006, 2007). Over time this can lead to structural and functional changes in these regions, in addition to increased behavioral withdrawal, which can further aggravate feelings of loneliness. Research has linked increased perceptions of loneliness to cognitive functions regulated by the amygdala, PFC, and hippocampus, including executive function and memory (Shankar et al., 2013; Cacioppo et al., 2014). Moreover, recent evidence suggests structural changes to the brain and cognitive impairments may precede increased perceptions of loneliness in older adulthood (Ayalon et al., 2016).

The purpose of this study was to examine the effects of changes in perceived social support and stress on change in loneliness after a 6-month, group-based exercise intervention. We hypothesized that increases in perceived social support over the course of the intervention would predict reductions in perceived loneliness, and that reductions in stress would partially mediate this effect (H1). We also examined interactions between these effects and changes in regional brain volumes (i.e., amygdala, PFC, hippocampus). Specifically, we tested three hypotheses of the role of brain volume within this framework. (1) Regional brain volumes may change over the course of the intervention and mediate the effects of changes in social support and stress on change in loneliness (H2). (2) Individuals with larger amygdala volumes at baseline may be more vulnerable to loneliness and stress and may, in turn, report greater reductions in loneliness after the intervention (H3). (3) Individuals with larger PFC and hippocampal volumes at baseline may have greater capacity for behavioral regulation and may, in turn, reported greater reductions in loneliness after the intervention (H4).

## Materials and methods

### Participants

**Figure 1:**
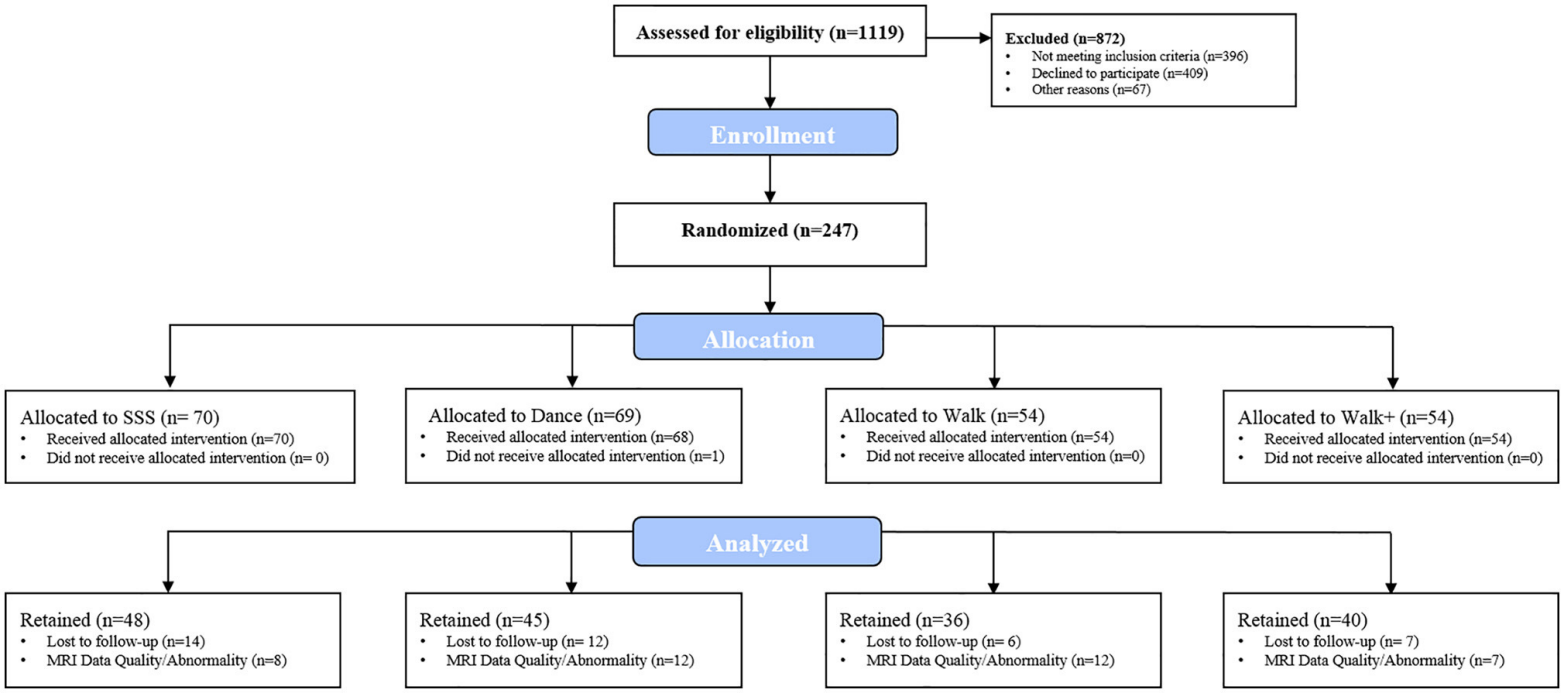
**Flow of participants**.

Participants were 247 community-dwelling older adults (mean age = 65.39 ± 4.56 years, 68.4% female) enrolled in a 24-week randomized controlled exercise trial examining the effects of aerobic exercise and cognitive training on brain health. Individuals were eligible to participate if they met the following inclusion criteria: (a) 60–79 years-old; (b) able to read and speak English; (c) right-handed; (d) low-active or inactive (i.e., participated in 30 or minutes of moderate physical activity fewer than 2 days per week over the past 6 months); (e) local to the study location for the duration of the program; (f) willing to be randomized to one of four interventions; (g) not involved in another physical activity program; and (h) scored >21 on the Telephone Interview of Cognitive Status questionnaire (de Jager et al., 2003) and >23 on the Mini Mental State Exam (Folstein et al., 1975). Eligibility also included meeting inclusion criteria for completing a magnetic resonance imaging (MRI) assessment, consisting of: (a) free from neurological disorders; (b) no history of stroke, transient ischemic attach, or surgeries including the removal of brain tissue; (c) no implanted devices or metallic bodies above the waste; (d) normal or corrected-to-normal vision of at least 20/40 in both eyes; and (e) no color blindness. The project coordinator assessed all interested individuals for eligibility via a telephone pre-screening interview. The flow of participants through the study is illustrated in Figure 1. Due to missing MRI data, 78 participants had incomplete data at baseline and post-intervention. Analyses were completed with the intent-to-treat principle, maintaining the complete sample and handling missing data via full information maximum likelihood—a non-imputation approach that leverages all available data during latent model estimation (Muthén et al., 1987; Little, 2013). This study was approved by and carried out in accordance with the recommendations of the Institutional Review Board at the University of Illinois at Urbana-Champaign with written informed consent from all participants. All participants provided written informed consent in accordance with the Declaration of Helsinki.

### Procedures

After all baseline data were collected, eligible participants were randomly assigned to one of four interventions implemented over four waves from October 2011 to November 2014: Dance (*n* = 69), Strength/Stretching/Stability (SSS; *n* = 70), Walk (*n* = 54), and Walk Plus (*n* = 54). Dance represented the aerobic + cognitive training condition, Walk and Walk Plus represented aerobic training only, and SSS served as the active, non-aerobic control condition. Each wave included ~18 participants per group. The trial was powered for the clinical trial's primary outcomes based upon MRI data from previous studies (Kramer et al., 1999; Colcombe and Kramer, 2003). Participants were randomized using a computer data management system and baseline-adaptive randomization scheme (Begg and Iglewicz, 1980). Participants in all conditions attended three 1-h exercise sessions per week for 24 weeks (~6 months). Each group session was supervised by trained exercise leaders, began with a brief warmup consisting of walking and full-body stretching, and concluded with an abbreviated set of stretches. The trial is registered with United States National Institutes of Health ClinicalTrials.gov (ID: NCT01472744).

### Training protocol

#### Dance

Individuals assigned to the Dance condition participated in social dancing comprised of American and English folk dancing. Participants learned three to five dances at the start of each month, with the speed and complexity of dances increasing over the course of each month and across the intervention. Dances were led by an experienced “caller,” who provided instruction on upcoming figures and reduced instruction over the course of each month.

#### Strength/stretching/stability (SSS)

Individuals assigned to the SSS condition participated in exercise sessions designed to improve flexibility, strength, and balance with the aid of yoga mats and blocks, chairs, and resistance bands. Sessions included 10–12 exercises and were led by trained exercise leaders. Participants learned new exercises at the start of each month. Exercises increased in complexity, difficulty, and volume over the course of each month and across the intervention. Three exercises leaders demonstrated different versions of each exercise throughout each session: the intended exercise, an easier modification of the exercise, and a more challenging version of the exercise.

#### Walk and walk plus

Individuals assigned to the Walk and Walk Plus conditions participated in walking sessions led by trained exercise leaders. Individuals assigned to Walk Plus also received a nutritional supplement containing antioxidants, anti-inflammatories, vitamins, minerals, and beta alanine (Abbott Nutrition, Abbott Park, Illinois). Preliminary analyses of the primary outcomes indicated no differences between the walking interventions; therefore, Walk and Walk Plus were collapsed for the present analyses. Participants walked at 50–60% of their maximal heart rate, as ascertained from a maximal graded exercise test and increased from 20 to 40 min of walking during the first 6 weeks of the program. During the remaining 18 weeks, participants walked for 40 min at 60–75% of their maximal heart rate each session.

### Measures

#### Perceived loneliness

Perceived loneliness was measured using the UCLA Loneliness Scale (Russell et al., 1980), a 20-item scale assessing the frequency with which individuals experience feelings of loneliness. Individuals are asked to respond to 9 positively and 11 negatively worded statements to indicate how often they feel, on a scale from 1 (never) to 4 (always), the way described by the statement. Positively worded items are reversed coded and all items are summed for a possible range of 20–80, with higher scores indicating greater loneliness. Reliability and validity of the UCLA Loneliness Scale has been previously established in older adults (Cutrona and Russell, 1986).

#### Perceived social support

Perceived social support was measured using the Social Provisions Scale (SPS) (Cutrona and Russell, 1987), a 24-item scale assessing relational provisions including: attachment, social integration, reassurance of worth, reliable alliance, guidance, and opportunity for nurturance. Each provision is assessed by four items, two describing the presence and two describing the absence of the provision. Items were modified for the exercise setting (e.g., “I feel part of the exercise group”). For each item participants respond in relation to the extent to which each statement described their current social relationships within an exercise program on a 4-point Likert scale from 1 (strongly disagree) to 4 (strongly agree).

#### Perceived stress

Perceived stress was measured using the Perceived Stress Scale (PSS) (Cohen et al., 1983; Cohen and Williamson, 1988), a 10-item scale assessing the degree to which an individual perceived life situations as stressful. Participants are asked to rate how often they feel a certain way (e.g., “nervous and stressed”), with response options ranging from 0 (never) to 4 (very often).

#### Regional brain volume

We acquired Magnetic Resonance (MR) images during a single session on a 3T Siemens Trio Tim (Siemens, Erlangen, Germany) to measure amygdala, PFC, and hippocampus volumes. High-resolution structural MR scans were acquired using a 3D MPRAGE T1-weighted sequence (TR = 1900 ms; TE = 2.32 ms; TI: 900 ms; flip angle = 9°; matrix = 256 × 256; FOV = 230 mm; 192 slices; resolution = 0.9 × 0.9 × 0.9 mm; GRAPPA acceleration factor 2). All images were individually checked for quality by AZB. Automated brain tissue segmentation and reconstruction of cortical models was performed on T1-weighted images using the Freesurfer software, version 5.3 (http://surfer.-nmr.mgh.harvard.edu/). In brief, individual T1-weighted images underwent non-brain tissue removal, Talairach transformation, creation of representations of the gray/white matter boundaries (Dale and Sereno, 1993; Dale et al., 1999), and calculation of the cortical thickness as the distance between the gray/white matter boundary and the pial surface at each point across the cortical mantle (Fischl and Dale, 2000). Surface reconstructions were carefully screened to evaluate the success and plausibility of the automatically processed results, as recommended by the software developers. Next, cortical thickness maps were inflated (Fischl et al., 1999a), smoothed using a circularly symmetric Gaussian kernel across the surface with a full width at half maximum of 10 mm, and registered to a spherical atlas to match individual cortical folding patterns across subjects (Fischl et al., 1999b). The mean cortical thickness of each cortical parcellation was then extracted and saved in text format for data analysis. Prior to data analysis regional brain volumes in each hemisphere were corrected for intracranial volume separately by time point (baseline, 6 months) via analysis of covariance (Jack et al., 1989). Volumes were corrected separately for males and females if the homogeneity of slopes assumption between sexes was violated.

### Data analytic strategy

Hypotheses were tested in a latent modeling framework estimated in MPlus software (v7; Muthén and Muthén). Changes in psychosocial and brain measures from pre- to post-intervention were estimated in latent change score models (LCSMs). A LCSM is similar to a difference score, but because it is derived from latent constructs, the mean estimate of change and individual differences therein are free of measurement error (McArdle and Nesselroade, 1994; McArdle, 2009). Latent constructs were defined by multiple measures: even and odd items of the UCLA scale for loneliness and the PSS scale for stress, and social support was defined by the attachment (emotional support), guidance (informational support), and reliable alliance (tangible aid) subscales. For each construct, a single indicator was fixed to 1 to identify the scale, the other identifying indicators were estimated, and all measurement residuals were estimated, which produced latent scores similar to error-free composites. To ensure the assumption of measurement invariance was met, several constraints were placed on the model: estimated factor loadings and measurement residuals were constrained to be equal across time, and measurement intercepts were fixed to 0. In addition, repeated measures were allowed to correlate across time. There were a few exceptions: the measurement residuals of the PSS were not constrained to be equal and even items were uncorrelated over time, nor were even items of the UCLA scale correlated, and residuals of left amygdala were freely estimated across timepoints. A similar procedure was applied in the grouped modeling, in which measurement invariance was imposed across time, as well as between groups, and only the mean effects, corresponding variance, and paths tested for group differences were freely estimated. Planned pair-wise comparisons between intervention conditions and the control group were made simultaneously during model estimation.

Prior to model estimation, all measures were normed to baseline means and standard deviations of the total sample. Thus, change scores can be interpreted as standardized change from baseline. Unless otherwise stated, all reported effects are unstandardized coefficients. A standardized effect size of mean latent change is reported: d = (Mean Latent Change)/√(Baseline Latent Variance). To test mediation hypotheses, parallel LCSMs were constructed (H1 and H2). A significant indirect effect was accepted as sufficient evidence of mediation (James and Brett, 1984). To avoid spurious results due to a smaller sample size, all LCSMs were bootstrapped with bias-correction (5,000 draws) (Hayes and Scharkow, 2013) to produce 95% confidence intervals (BS 95% CI) of unstandardized effects. Latent models included the complete sample and missing data were handled via full information maximum likelihood (FIML)—a non-imputation approach that leverages all available data during effect estimation (Muthén et al., 1987; Larsen, 2011), and the current recommended practice for longitudinal studies with attrition(Little, 2013). Model fit was determined by several accepted indices (Hu and Bentler, 1999): non-significant normal theory weighted chi-square (χ^2^), comparative fit index (CFI > 0.90), root mean square error of approximation (RMSEA < 0.05), and standardized root mean residual (SRMR < 0.08). Model fit was determined for the total sample and with grouped modeling procedures.

To test the hypothesis that baseline brain volumes may moderate the relationship between changes in social support and stress predicting change in loneliness (H3 and H4), models were tested in a random effects framework. In this modeling approach, path moderation is tested by calculating an interaction term between two latent constructs (i.e., brain volume × change in stress) and the path of the dependent variable regressed on the interaction is tested for significance. The sample was split at the median latent score for each regional brain volume to test the moderation effects. Due to the modeling approach, model fit can only be evaluated by nested model comparisons with Akaike information criterion (AIC) and sample-size adjusted Bayesian information criterion (BIC), for which lower values indicated relatively improved fit. The hypothesized moderation models were compared to constrained models that estimated the interaction terms but did not predict outcome measures. Thus, relative improvement in this comparison suggests the hypothesized moderation effect fits the observed data better than no effect. For random effects models, 95% confidence intervals (95% CI) of unstandardized effects without bootstrapping procedures are reported.

## Results

### Descriptive summary of sample

A descriptive summary of the sample is provided in Table 1. Briefly, our sample was comprised primarily of females, and white, college educated, and married older adults. Descriptive statistics of manifest psychosocial variables are reported in Table 2. One-way analysis of variance comparing participants in each exercise condition indicated that participants across the four conditions did not differ in demographics, psychosocial variables, or regional brain volumes at baseline (all *p* > 0.05).

**Table 1:** **Sample characteristics**.

	***N***	**(%)**
	**M**	**±SD**
Female	169	(68.4)
Body Mass Index (kg/m^2^)	30.98	±5.58
**RACE**		
White	207	(83.8)
African American	32	(13.0)
Asian	8	(3.2)
**EDUCATION**		
Non-college graduate	102	(41.3)
College graduate	145	(58.7)
**MARITAL STATUS**		
Married	146	(59.1)
Partnered	6	(2.4)
Single	30	(12.1)
Divorced/Separated	36	(14.6)
Widowed	29	(11.7)

*M, Mean; SD, Standard Deviation*.

**Table 2:** **Descriptive summary of psychosocial variables**.

	**Baseline**	**Month 6**
Social Support		
Total Score^*^	83.11 ± 9.67	84.82 ± 9.13
Attachment^*^	13.71 ± 2.26	14.06 ± 2.22
Guidance	14.18 ± 2.07	14.48 ± 1.99
Reliable Alliance	14.58 ± 1.90	14.81 ± 1.77
Perceived Stress^*^	11.96 ± 6.11	10.57 ± 5.83
Loneliness^*^	37.12 ± 9.77	35.31 ± 8.91

* *p < 0.01, Baseline to Month 6*.

### Longitudinal consistency of measures

Prior to latent modeling, the internal consistency of the measures within persons over time was evaluated with Pearson correlations. Stress indices (PSS) were divided into even and odd items for the purpose of latent construction, and these were moderately consistent over the course of the intervention (*r* = 0.64 and 0.57, respectively). Measures of social support had relatively high internal consistency with the attachment subscale having the greatest (*r* = 0.78) and the alliance subscale the least (*r* = 0.59). The items of the UCLA scale for loneliness had a similar degree of consistency (*r* = 0.65 and 0.69 for even and odd items, respectively). Finally, volume measures of amygdala (*r* = 0.71 and 0.77), PFC (*r* = 0.84 and 0.88), and hippocampus (*r* = 0.81 and 0.86) were highly consistent over time. The degree of inconsistency that remained in the measures was accounted for in the LCSMs by constraining factor loadings, measure residuals, and intercepts to be the same across time, thereby imposing the assumption of measurement invariance during hypothesis testing.

### Change in social support, stress, and loneliness (H1)

Estimated as correlated latent change scores, subjective reports of social support increased (mean = 0.12, *p* = 0.003), whereas stress (mean = −0.20, *p* < 0.001) and loneliness (mean = −0.16, *p* = 0.001) decreased over the course of the intervention. Further, individuals significantly varied in the magnitude of change in each of these constructs (see Table 3), suggesting possible covariates to change. Persons with higher levels of stress (−0.24, *p* < 0.001) and loneliness (−0.16, *p* < 0.001) at baseline demonstrated greater decreases over the course of the intervention, but baseline scores of social support were unrelated to change within the construct (−0.03, *p* = 0.18).

**Table 3:** **Mean latent change and individual differences in psychosocial scores**.

**Latent construct**	**Baseline**	**Change**			
	**Variance**	**Mean**	**[BS 95% CI]**	**Variance**	***d***
Stress	0.97^*^	−0.20^*^	[−0.29/−0.10]	0.55 ^*^	−0.20
Loneliness	0.63^*^	−0.15^*^	[−0.23/−0.08]	0.44 ^*^	−0.19
Social support	0.84^*^	0.11^*^	[0.05/0.18]	0.19 ^*^	0.12

Estimates were taken from a correlated, latent change score model. Unstandardized coefficients are reported.

* *p < 0.05, BS 95% CI—bias-corrected bootstrapped 95% confidence intervals. d is a standardized effect size for latent mean change: d = (Mean Change)/√(Baseline variance)*.

**Figure 2:**
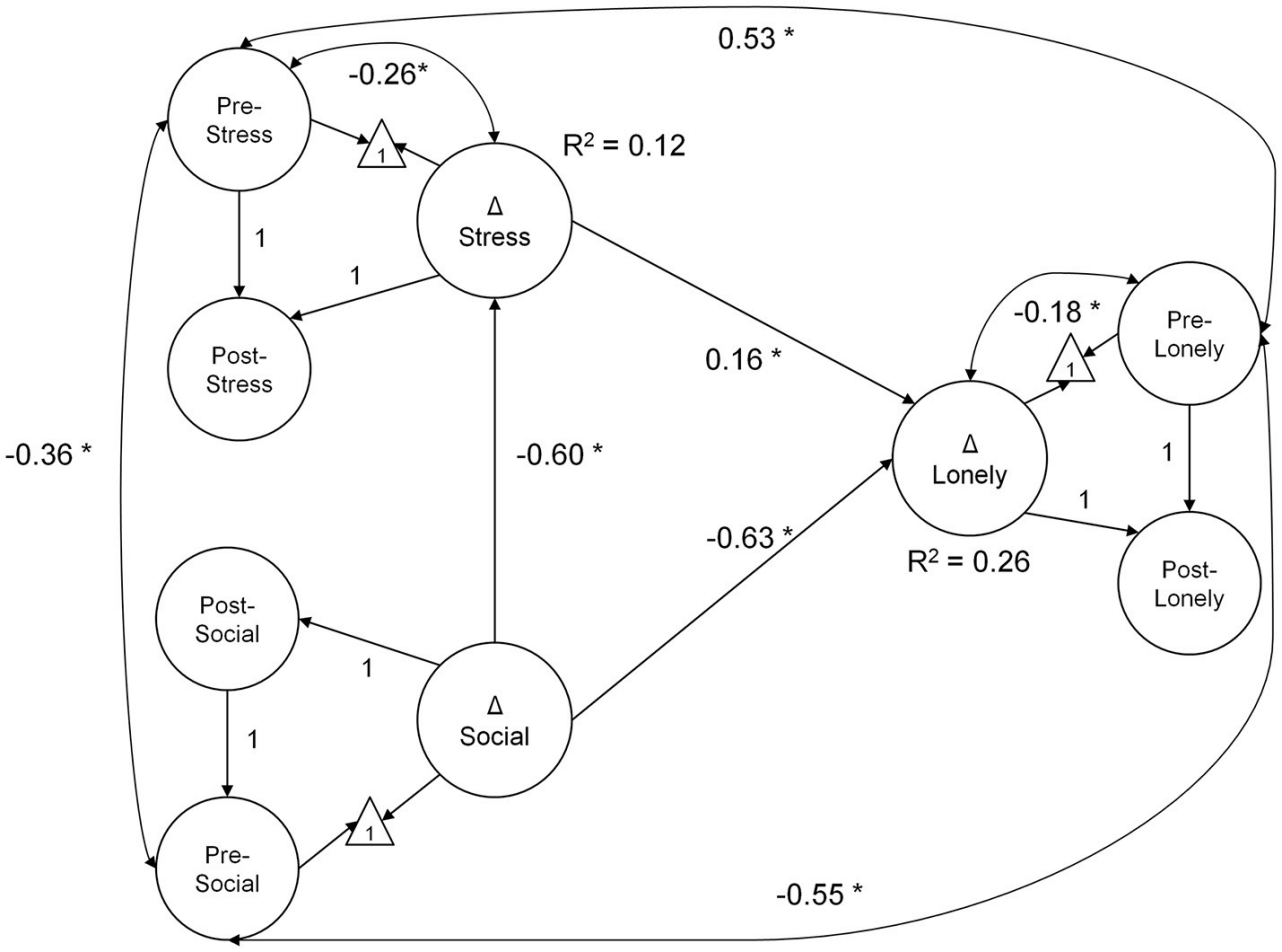
**Mediation model diagram of latent change in social support, stress, and loneliness**. Greater decline in loneliness scores over the course of the intervention were explained by decreases in stress and increased social support. Further, the effect of gains in social support was mediated by decreased stress (indirect effect = −0.10, *p* = 0.02; BS 95% CI: −0.21/−0.03). All coefficients are unstandardized, estimated from data normed to baseline scores. The measurement model is not illustrated (see description in Methods section); latent paths fixed to 1 were used to identify the latent change score. In the model diagram, straight arrows represent regression paths, curved double-headed arrows, correlations. ^*^p < 0.05; Δ—latent change score.

In the mediation model testing H1 (Figure 2), greater decrease in stress explained greater reductions in loneliness (0.16, *p* = 0.01; BS 95% CI: 0.04/0.29). Moreover, increased social support was directly related to decreased loneliness (−0.63, *p* < 0.01; BS 95% CI: −0.84/−0.35). An indirect effect of increased social support, mediated by decreased stress, was also observed (indirect effect = −0.10, *p* = 0.02; BS 95% CI: −0.21/−0.03). Collectively, changes in social support and stress explained ~26% of the individual variability in change in loneliness. The mediation model had excellent fit: $\chi_{\left(81\right)}^{2}$ = 100.27, *p* = 0.07, CFI = 0.99, RMSEA = 0.03, SRMR = 0.06. The model of correlated change fit similarly well: $\chi_{\left(80\right)}^{2}$ = 103.09, *p* = 0.04, CFI = 0.99, RMSEA = 0.03, SRMR = 0.06.

### Changes in regional brain volumes as mediators of change in loneliness (H2)

Amygdala volume did not change over the course of the intervention (mean = 0.04, *p* = 0.40; BS 95% CI: −0.04/0.14; d = 0.05), whereas PFC volume decreased (mean = −0.09, *p* = 0.03; BS 95% CI: −0.17/−0.02; d = −0.10) and a trend for the same in the hippocampus failed to reach significance (mean = −0.07, *p* = 0.07; BS 95% CI: −0.12/−0.01; d = −0.08). Individuals significantly varied in their magnitude of change in volume of amygdala (variance = 0.21, *p* < 0.001), PFC (variance = 0.25, *p* < 0.001), and hippocampus (variance = 0.11, *p* = 0.001). Yet, change in amygdala (all *r* = 0.02 to −0.02, *p* ≥ 0.44), PFC (all *r* = −0.02 to 0.00, *p* ≥ 0.52), and hippocampus (all *r* = −0.02 to 0.01, *p* ≥ 0.42) were unrelated to changes in social support, stress, or loneliness. Thus, concurrent changes in brain volumes were unrelated to psychosocial outcomes, providing no evidence in support of H2. The models of change in amygdala and PFC volume had good fit (respectively): $\chi_{\left(5,\;3\right)}^{2}$ = 11.17 and 3.15, *p* ≥ 0.05, CFI ≥ 0.99, RMSEA = 0.09 and 0.02, SRMR = 0.06 and 0.10; and the model of change in hippocampal volume had lesser fit: $\chi_{\left(5\right)}^{2}$ = 26.95, *p* < 0.001; CFI = 0.97; RMSEA = 0.16, SRMR = 0.04. Finally, the model including correlated changes of brain volumes with social support and affective measures fit well: $\chi_{\left(283\right)}^{2}$ = 341.08, *p* = 0.01, CFI = 0.99, RMSEA = 0.03, SRMR = 0.06.

### Baseline regional brain volumes as moderators of change in loneliness (H3 and H4)

The following correlations were accounted for in the model that tested baseline brain volumes as moderators of the effects of changes in stress and social support on change in loneliness (H3 and H4). At baseline larger amygdala volume correlated with lower perceived social support (−0.15, *p* = 0.01) and greater loneliness (0.14, *p* = 0.01), but not with stress (0.11, *p* = 0.05). PFC (all *r* = −0.13 to 0.12, *p* ≥ 0.05) and hippocampal volumes (all *r* = −0.08 to −0.06, *p* ≥ 0.15) were unrelated to social and affective measures at baseline. Baseline amygdala volume was not directly correlated with changes in any measure (all *r* = 0.002 to 0.09, all *p* ≥ 0.08). Larger baseline PFC volume was correlated with greater reductions in loneliness (*r* = 0.12, *p* = 0.01), but not change in social support (*r* = −0.03, *p* = 0.49), or stress (*r* = 0.03, *p* = 0.43).

**Figure 3:**
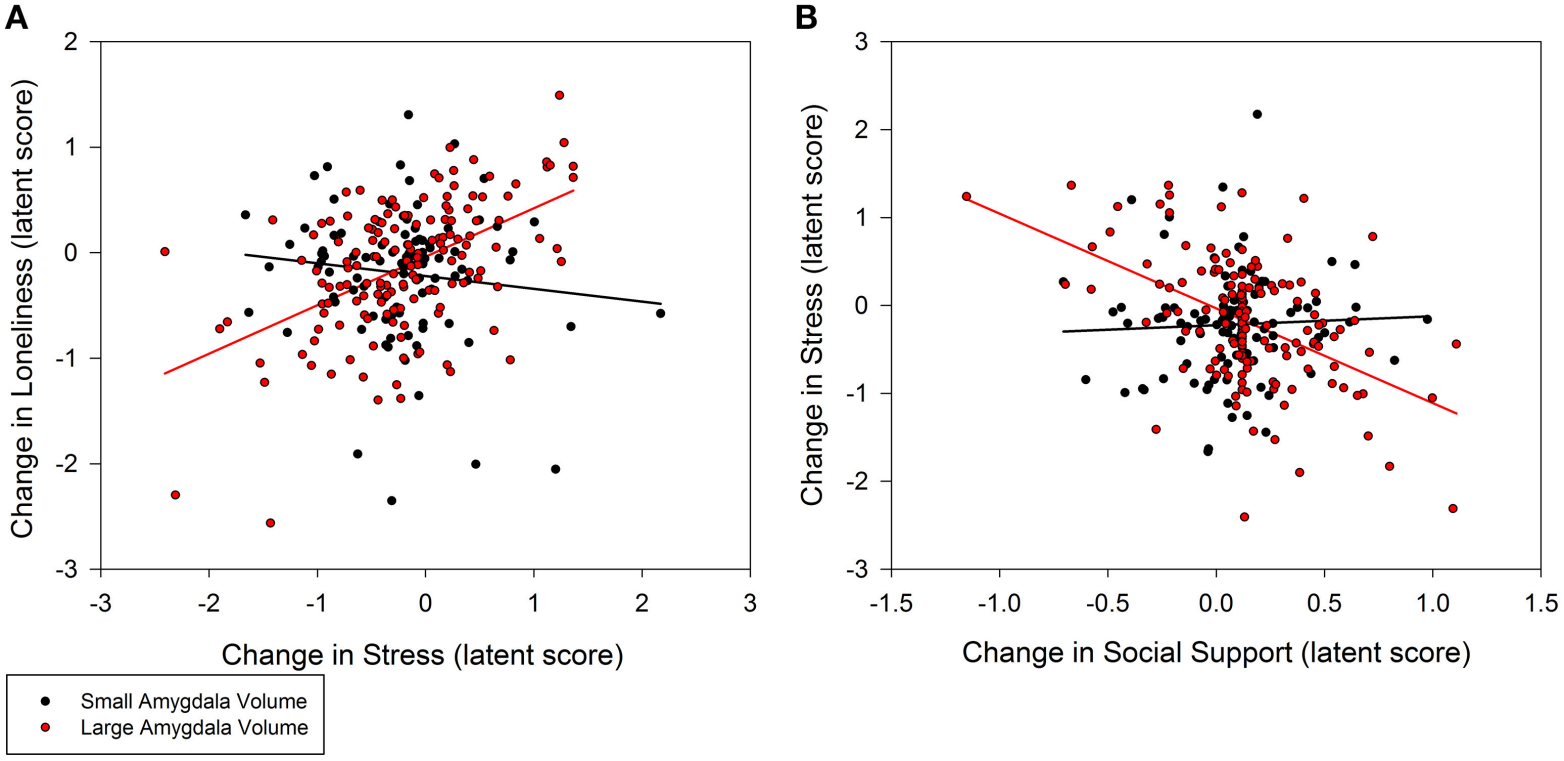
**Moderation of change in stress and social support predicting change in loneliness by baseline amygdala volumes**. **(A)** Persons with larger amygdala volumes at baseline experienced greater decrease in stress that explained greater decrease in loneliness (0.35, *p* = 0.02; 95% CI: 0.10/0.60). **(B)** Larger amygdala volumes also explained greater gains in social support that in turn accounted for greater declines in stress (−0.48, *p* = 0.08; 95% CI: −0.94/−0.02), and subsequently declines in loneliness. Amygdala volume was treated as a continuous, latent score in a random effects model; the sample was split at the median latent score to illustrate the moderation effect.

**Figure 4:**
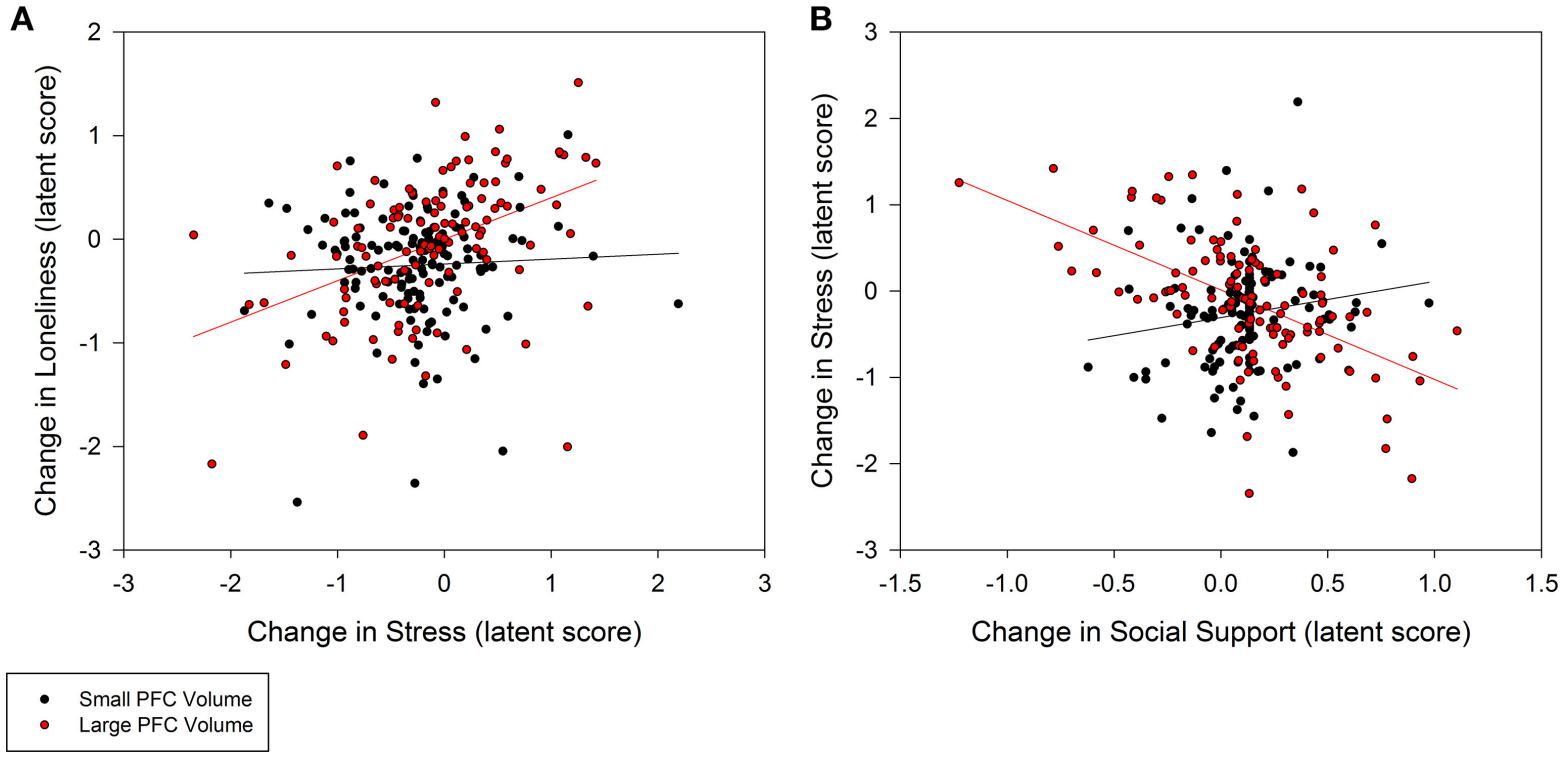
**Moderation of change in stress and social support predicting change in loneliness by baseline prefrontal cortex (PFC) volumes**. **(A)** Persons with larger PFC volumes at baseline experienced greater decrease in stress that explained greater decrease in loneliness (0.30, *p* = 0.02; 95% CI: 0.08/0.52). **(B)** Larger PFC volumes also explained greater gains in social support that in turn accounted for greater declines in stress (−0.28, *p* = 0.05; 95% CI: −0.51/−0.05), and subsequently declines in loneliness. PFC volume was treated as a continuous, latent score in a random effects model; the sample was split at the median latent score to illustrate the moderation effect.

Individuals with larger baseline amygdala volumes were more likely to experience a greater decrease in loneliness from stress reduction over the course of the intervention (0.35, *p* = 0.02; 95% CI: 0.10/0.60; Figure 3A). A non-significant trend further indicated that larger amygdala volume accounted for greater stress reduction due to increases in social support (−0.48, *p* = 0.08; 95% CI: −0.94/−0.02; Figure 3B). Similarly, individuals with larger PFC volume experienced greater reduction in loneliness which was directly related to decreased stress (0.30, *p* = 0.02; 95% CI: 0.08/0.52; Figure 4A). A non-significant trend was also observed in which larger baseline PFC volume modified the effect of increases in social support on reductions in stress (−0.28, *p* = 0.05; 95% CI: −0.51/−0.05; Figure 4B). Neither amygdala (−0.12, *p* = 0.65; 95% CI: −0.55/0.31) nor PFC volume (−0.01, *p* = 0.98; 95% CI: −0.29/0.28) modified the direct path from change in social support to change in loneliness. The hypothesized models fit better than the nested model comparisons that constrained the moderation paths for volume measures of amygdala (AIC = 7216.49; BIC = 7231.76 vs. AIC = 7222.93; BIC = 7236.17) and PFC (AIC = 7048.79, BIC = 7063.38 vs. AIC = 7066.39, BIC = 7079.62). Individual differences in baseline hippocampal volume did not modify changes in loneliness from stress reduction (−0.05, *p* = 0.60; 95% CI: −0.21/0.11) or increased social support (0.24, *p* = 0.31; 95% CI: −0.15/0.63); however, a non-significant trend for greater reduction in stress from social support was observed (0.40, *p* = 0.07; 95% CI: 0.04/0.76). The hypothesized model fit including hippocampal volumes (AIC = 7156.01, BIC = 7171.28) was similar to the constrained model (AIC = 7157.90, BIC = 7171.14), further suggesting a negligible influence of hippocampal volume on the relationship among social support, stress, and loneliness.

### Reverse effects

To further test the direction of effects in the hypothesized model, we tested a reverse effects model that inverted the main paths between loneliness, stress, and social support. The reverse effects model fit incrementally worse than the hypothesis model [$\chi_{\left(81\right)}^{2}$ = 111.64, *p* = 0.01, CFI = 0.99, RMSEA = 0.04, SRMR = 0.06], and change in loneliness did not directly predict change in stress (0.14, *p* = 0.20). However, decreases in loneliness explained increases in social support (−0.27, *p* < 0.001) that, in turn, accounted for decreases in stress (−0.44, *p* = 0.03; indirect effect = 0.14, *p* = 0.04; BS 95% CI: 0.04/0.31; *R*^2^ = 0.11). While the hypothesized model is substantiated in theory and supported by model fit, the direction of the paths should be interpreted with caution.

### Group differences

Finally, possible group differences in mean latent change and in the mediation paths were tested using a grouped modeling procedure. The intervention conditions experienced similar change in loneliness (difference = −0.03 to 0.18, *p* ≥ 0.15), social support (difference = −0.03 to 0.08, *p* ≥ 0.45), and stress (difference = −0.22 to −0.12, *p* ≥ 0.29), and the magnitude of all paths in the mediation model were similar to the SSS group (i.e., control group; all *p* ≥ 0.11). The grouped mediation model had sub-optimal fit indices: $\chi_{\left(354\right)}^{2}$ = 420.45, *p* = 0.01, CFI = 0.98, RMSEA = 0.06, SRMR = 0.11. Therefore, the mode of exercise intervention did not account for individual differences observed in changes in social support, stress, and loneliness.

## Discussion

This is one of the first studies to examine interactions among psychosocial and neural predictors of loneliness in the context of an intervention. Similar to previous research, increases in social support due to participation in an exercise intervention directly predicted reductions in loneliness (McAuley et al., 2000) as well as indirectly through decreases in perceived stress. These effects were consistent across exercise group assignment, suggesting exercise mode may not be a factor in older adults' perceptions of loneliness (McAuley et al., 2000; Mackenzie et al., 2014). Contrary to our hypothesis, the influence of changes in social support and stress on loneliness were not related to change in brain volumes. However, larger amygdalae at baseline accounted for greater decreases in loneliness amidst reductions in stress and larger PFC volumes accounted for greater decreases in stress amidst increases in social support, but neither directly modified the response in loneliness to changes in social support. Taken together these findings suggest potential target mechanisms to reduce loneliness and have important implications for physical activity recommendations for health benefits in aging adults.

While previous studies have observed direct effects of increases in social support on reductions in loneliness after exercise interventions (McAuley et al., 2000; Hwa Kwag et al., 2011), our findings indicate that the impact of social support on feelings of loneliness may also be indirect through decreases in perceived stress. Mode of exercise did not alter the pattern of changes, suggesting group-based exercise programs, in general, may provide these benefits. This is similar to recent evidence from McHugh and Lawlor (2011) in which exercise and social support independently influenced psychological distress (depression, anxiety, and stress) in older adults. The authors concluded that the benefits of exercise on psychological outcomes may be partially related to social interactions inherent to group-based programs. Together these results indicate a need for further research comparing social and non-social (e.g., home-based) exercise programs and dissecting the components of social exercise programs that contribute to reductions in loneliness. Our results, along with previous studies (McAuley et al., 2000; Mackenzie et al., 2014), suggest factors other than mode of exercise may explain relationships among social support, stress, and loneliness. This is further supported by the lack of effects in relation to change in regional brain volumes. Although individuals varied in the magnitude of change, changes in social support, stress, and loneliness were unrelated to individual differences in brain volume changes, nor did these mediate the concurrent psychosocial changes. Aerobic activity and resulting improvements in cardiorespiratory fitness are known to increase PFC and hippocampal volumes in older adults (Kramer et al., 1999; Colcombe et al., 2006; Erickson et al., 2011; Voss et al., 2013). Because improvements in social support, stress, and loneliness occurred across groups and in the absence of improvements in brain volumes, reductions in loneliness after exercise programs may not be restricted to aerobic exercise or be the result of exercise's effects on brain structure. Further research in this area may help us to better understand the theoretical pathway between exercise participation and loneliness.

Despite this previous evidence, the lack of mediation effects related to change in brain volumes may in part be due to the relatively short time period and generally high-functioning nature of the sample of older adults. Older age is typically associated with robust shrinkage of the PFC and hippocampus that is exacerbated by age-related decline in cardiovascular health and carriage of genetic pro-inflammatory risk (Raz and Rodrigue, 2006; Persson et al., 2014). Aerobic exercise is expected to mitigate these risks during aging to promote better maintenance of brain volumes into senium, with some studies even reporting volumetric gains in healthy older adults after 6 months of exercise (Kramer et al., 1999). Here we observed decreases in PFC and a trend for the same in hippocampal volumes. However, individuals varied in the magnitude of change, suggesting factors in the course of study may have promoted maintenance of brain structure. Indeed, the magnitude of shrinkage observed in these regions (PFC d = −0.10; hippocampus d = −0.08) were smaller than other longitudinal studies of brain aging (Driscoll et al., 2009; Persson et al., 2014), further suggesting a protective influence of exercise on the brain. Despite this, change or relative stability of regional brain volumes over 6 months did not account for concurrent changes in psychosocial measures. The impact of the intervention on brain volume may have been too small during the relatively short period to detect association with reductions in loneliness. Interventions lasting 1 year or longer, and long-term follow-up assessments may be needed to better assess the potential mediating role of change in brain structure on change in loneliness (Cacioppo et al., 2003). Similarly, as these effects are expected to be larger in samples at higher health risk, exercise interventions targeting socially isolated older adults (Cacioppo et al., 2014) and those with mild cognitive impairment are also warranted.

Although we failed to find evidence of change in brain volumes interacting with concurrent change in psychosocial measures, baseline amygdala, and PFC volumes did account for individual differences in response to the intervention. Individuals with larger amygdalae at baseline were more likely to report greater reductions in loneliness following decreases in stress. This was related, in part, to increases in social support. Amygdala volume was also negatively correlated with perceived social support at baseline. Therefore, older adults with larger amygdalae may have gained more from the social support environment of the exercise sessions, particularly as demonstrated by a decline in perceived stress. Amygdala volume has been linked to anxiety and depression, and an overactive amygdala can result in impaired cognitive control processes in other areas of the brain, particularly the PFC (Davidson and McEwen, 2012).

Similarly, increased PFC activation amidst decreased negative affect exerts an inhibitory influence on amygdala activation (Urry et al., 2006). The PFC is involved in behavioral and emotional regulation, attentional and inhibitory control, and response selection (Miller and Cohen, 2001). Functional deficits in the PFC can therefore impair the regulation of behaviors such as physical activity and emotions such as stress, thereby potentially exacerbating perceptions of loneliness (Hawkley and Cacioppo, 2007). Individuals with better cognitive control, on the other hand, may be better able to regulate amygdala activation and negative emotions (Urry et al., 2006; Etkin et al., 2011). Consistent with this evidence, individuals with larger baseline PFC volumes were more likely to report greater reductions in stress following increases in social support. Interestingly, the influence of reductions in stress on decreases in loneliness were strongest for participants with larger amygdalae. Because participants in the current study were low-active and less familiar with regular exercise, those with large PFC volumes may have exerted more cognitive control and more easily adapted to the three-times weekly exercise intervention (Miller and Cohen, 2001). Amygdala results suggest these adaptations may have been most important for older adults with chronic stress. Further research to identify the PFC- and amygdala-related cognitive processes (e.g., self-regulation, inhibitory control, and executive function) that may be associated with changes in social support, stress, and loneliness may help to further explain affective responses to exercise programs. For example, McAuley et al. (2011) observed direct and indirect effects (via self-efficacy) of PFC-dependent cognitive functions, including self-regulation and executive function, on older adults' adherence to an exercise program. Additional examination of neural and cognitive predictors of exposure to exercise interventions may inform the design of future interventions that optimize older adults' behavioral and affective response to exercise.

## Limitations

The present study is one of the first to examine interactions between brain structure and psychosocial health indicators and their influences on changes in loneliness after a behavioral intervention (Kanai et al., 2012; Cacioppo et al., 2014). We examined pathways of change in loneliness using a relatively large sample, a randomized experimental design, and sophisticated methods of data analysis. Despite these strengths, this study is not without limitations. First, as emotional regulation involves complex interactions among regional brain structure and function, it is possible that other brain regions (e.g., anterior cingulate cortex, striatum) may explain the relationships observed among social support, stress, and loneliness (Davidson and McEwen, 2012; Cacioppo et al., 2014). However, these preliminary findings support further investigation in this largely unexplored area of research. Additionally, while we accounted for participant attrition via FIML estimation, some bias may still be present, as over 30 percent of our sample had missing MRI data at baseline and/or post-intervention. Finally, we cannot definitively demonstrate causal lead-lag relationships among changes in perceived social support, stress, and loneliness. While the hypothesized model is supported by theory and fits the data well (Cohen, 2004; Holt-Lunstad and Uchino, 2015), the temporal order of effects remains equivocal across the loneliness literature (Hawkley and Cacioppo, 2010; Ayalon et al., 2016).

## Conclusions

Exercise programs, regardless of exercise mode, may be effective vehicles for reducing loneliness in older adults. The social support environment of exercise sessions and resulting declines in stress may at least partially explain these reductions. Older adults with larger amygdala and PFC volumes—regions implicated in processing emotional and social experiences—were more likely to report reductions in loneliness due to reductions in stress and gains in social support. Biomarkers of brain structural integrity, along with perceptions of social support and stress, may help researchers identify for whom an exercise intervention may be most effective. Given the nascence of this research, these preliminary findings warrant additional investigation of the mediating and moderating roles of brain structure on changes in loneliness as a result of participation in exercise programs. Studies comparing social and non-social exercise programs and examining other brain regions in addition to the amygdala, PFC, and hippocampus are specifically needed.

## Author contributions

DE defined the scientific investigation and was involved with program implementation, data analysis, and preparation of the manuscript. AD conducted the primary data analysis, and was involved with data interpretation and preparation of the manuscript. AB assisted in defining the scientific investigation, conducted primary processing of brain volumetric data, and was involved with the preparation of the manuscript. JF and EA were responsible for the implementation of the research and collection of psychosocial data, and were involved with the preparation of the manuscript. LC assisted in defining the scientific investigation. AK and EM are the senior authors who conceived the overall study, provided mentorship in defining the scientific investigation, and were involved in preparation of the manuscript.

## Funding

Preparation of this manuscript was supported by grants from the National Institute on Aging (R37 AG025667) and the Abbott Nutrition through the Center for Nutrition, Learning, and Memory at the University of Illinois at Urbana-Champaign. DE is supported by an American Cancer Society Postdoctoral Fellowship (PF-16-021-01-CPPB). AD is supported by a Beckman Institute Postdoctoral Fellowship at the University of Illinois at Urbana-Champaign, with funding provided by the Arnold and Mabel Beckman Foundation.

### Conflict of interest statement

The authors declare that the research was conducted in the absence of any commercial or financial relationships that could be construed as a potential conflict of interest.
